# Factors associated with unplanned readmissions and costs following resection of brain metastases in the United States

**DOI:** 10.1038/s41598-021-01641-4

**Published:** 2021-11-12

**Authors:** Raees Tonse, Alexandra Townsend, Muni Rubens, Vitaly Siomin, Michael W. McDermott, Martin C. Tom, Matthew D. Hall, Yazmin Odia, Manmeet S. Ahluwalia, Minesh P. Mehta, Rupesh Kotecha

**Affiliations:** 1 Department of Radiation Oncology, Miami Cancer Institute, Baptist Health South Florida, Office 1R203, Miami, FL 33176 USA; 2 Herbert Wertheim College of Medicine, Florida International University, Miami, FL USA; 3 Office of Clinical Research, Miami Cancer Institute, Baptist Health South Florida, Miami, FL USA; 4 Department of Neurosurgery, Miami Neuroscience Institute, Baptist Health South Florida, Miami, FL USA; 5 Division of Neuro-Oncology, Miami Cancer Institute, Baptist Health South Florida, Miami, FL USA; 6 Department of Medical Oncology, Miami Cancer Institute, Baptist Health South Florida, Miami, FL USA

**Keywords:** Cancer, CNS cancer

## Abstract

The purpose of this study was to critically analyze the risk of unplanned readmission following resection of brain metastasis and to identify key risk factors to allow for early intervention strategies in high-risk patients. We analyzed data from the Nationwide Readmissions Database (NRD) from 2010–2014, and included patients who underwent craniotomy for brain metastasis, identified using ICD-9-CM diagnosis (198.3) and procedure (01.59) codes. The primary outcome of the study was unplanned 30-day all-cause readmission rate. Secondary outcomes included reasons and costs of readmissions. Hierarchical logistic regression model was used to identify the factors associated with 30-day readmission following craniotomy for brain metastasis. During the study period, 44,846 index hospitalizations occurred for patients who underwent resection of brain metastasis. In this cohort, 17.8% (n = 7,965) had unplanned readmissions within the first 30 days after discharge from the index hospitalization. The readmission rate did not change significantly during the five-year study period (*p-trend* = 0.286). The median per-patient cost for 30-day unplanned readmission was $11,109 and this amounted to a total of $26.4 million per year, which extrapolates to a national expenditure of $269.6 million. Increasing age, male sex, insurance status, Elixhauser comorbidity index, length of stay, teaching status of the hospital, neurological complications and infectious complications were associated with 30-day readmission following discharge after an index admission for craniotomy for brain metastasis. Unplanned readmission rates after resection of brain metastasis remain high and involve substantial healthcare expenditures. Developing tools and interventions to prevent avoidable readmissions could focus on the high-risk patients as a future strategy to decrease substantial healthcare expense.

## Introduction

Approximately 10–30% of cancer patients develop brain metastasis during the course of their systemic disease, resulting in over 200,000 cases diagnosed annually in the United States^1^. There are multiple indications for resection of brain metastasis, including lengthening survival in a subset of patients, providing a pathological diagnosis, relieving tumor-related mass effect, improving neurologic functional status, and optimizing multi-modal management of large brain metastasis^2^. Approximately 1 in 6 patients who undergo a craniotomy is readmitted within 30 days of their index surgical admission at an estimated cost of $20,296 per readmission^3^. There has been a substantial reduction in the rates of unplanned readmission following many extracranial surgeries, however, single-institution studies have demonstrated relatively stagnant trends in neurosurgery readmission rates^3^. Current estimates theorize that approximately 70% of all-cause readmissions are avoidable. By establishing the factors that make hospital readmissions preventable after neurosurgical procedures, risk management for craniotomies will improve patient care while mitigating the related financial cost of the hospital readmission care^4^.

In an effort to decrease unplanned readmission rates for selected medical and surgical conditions, the Centers of Medicare & Medicaid Services (CMS) established the Hospital Readmissions Reduction Program (HRRP) within the Affordable Care Act in 2012^5^. As a result of this program, hospitals are penalized for increased rates of risk-standardized 30-day readmissions for pre-determined conditions, such as acute myocardial infarction, total hip and knee arthroplasty, chronic obstructive pulmonary disorder, and heart failure, with other conditions likely added in the future. After the implementation of the HRRP program, hospital systems have implemented various measures to decrease readmissions rates. Studies performed after the introduction of HRRP show many targetable risk factors, such as surgical volume, postoperative complications, and hospital length of stay, significantly affected the readmission rate after surgeries^6,7^.

Substantial studies on factors associated with readmission rates after craniotomy are yet unpublished. Understanding these elements could help lower readmission rates, reduce healthcare costs, as well as improve the quality of life of brain metastasis patients. The objective of this study was to develop a risk model for predicting 30-day unplanned readmission rates after surgical treatment of brain metastasis.

## Materials and methods

### Data source and design

This was a cross-sectional analysis of data collected from the Nationwide Readmissions Database (NRD) from 2010 to 2014. The Agency for Healthcare Research and Quality (AHRQ) began NRD as a component of the Healthcare Cost and Utilization Project (HCUP). NRD is a publicly accessible all-payer inpatient database that reports approximately 49% of all US hospitalizations from 21 states^8,9^. NRD contains discharge data from all US community hospitals, excluding rehabilitation centers and acute long-term facilities.

### Study population

**Figure 1:**
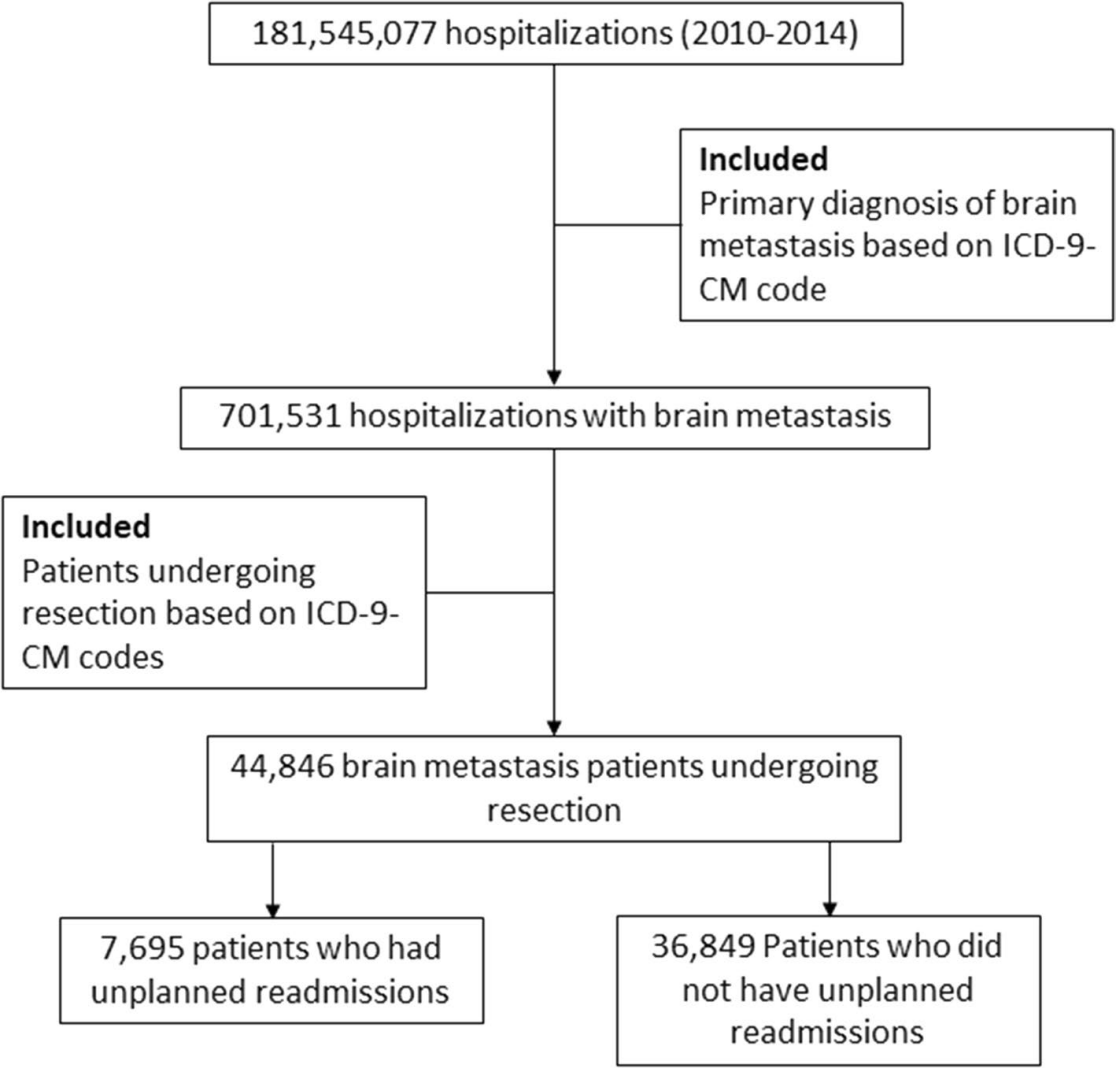
CONSORT diagram for inclusion criteria of the study.

We included data from all patients who underwent resection of brain metastasis. We identified patients undergoing craniotomies using ICD-9-CM diagnosis codes and the performed surgical procedures using ICD-9-CM procedure codes (Supplemental Table 1). We excluded patients who died during index hospitalizations, had planned readmissions, did not have information in discharge dispositions, or transferred to other acute care hospitals. We also excluded patients who were discharged in December due to the lack of 30-day follow-up data during the study period. Only first readmissions were included when patients experienced multiple readmissions within 30 days of discharge from the index hospitalization. Figure 1 shows the CONSORT diagram for participant inclusion in this study.

### Patient and hospital characteristics and surgical procedures

For each initial hospitalization, the NRD includes information regarding numerous patient variables. Patient characteristics abstracted included age, sex, primary payor, median household income for patient’s ZIP Code, Elixhauser comorbidity index, and length of stay. Hospital factors in NRD used in this study included hospital bed size, hospital teaching status (metropolitan and teaching vs. non-metropolitan), and hospital ownership.

### Outcomes measures

For this study, the primary outcome was unplanned 30-day all-cause readmission rates. The study used methods described by HCUP in order to identify all readmissions and the methods delineated by Horwitz et al. for unplanned readmissions^10^. We adhered to the Strengthening the Reporting of Observational Studies in Epidemiology (STROBE) guidelines for cohort studies while describing our findings^11^. The study was reviewed by the Miami Cancer Institute’s Institutional Review Board, which exempted the study from institutional review board approval and waived the requirement for informed consent because it uses previously collected deidentified data stored in NIS. Informed consent was not required since this study involves an administrative database and does not contain any identifiable information that can be linked to any specific participant. All the methods adhered to relevant ethical guidelines for handling human data.

### Statistical analysis

SAS, version 9.4 (Cary, NC) was used for statistical analyses. Complex survey weights and procedures were used for calculating national approximations. We used appropriate survey nature (SURVEYFREQ, SURVEYMEANS, and SURVEYGLIMMIX), weight (DISCWT), stratification (NRD_STRATUM), and clustering (HOSP_NRD) of the NRD to calculate national estimates. Baseline demographic, clinical, and hospital characteristics were compared between patients with and without unplanned 30-day readmissions. T-test and chi-square test were utilized for comparing continuous and categorical variables. We used hierarchical logistic regression model to identify the factors associated with 30-day readmission following craniotomy for brain metastasis since it accounts for the effect of nesting, where patient-level effects are nested with hospital-level effects. In addition, hospital identification number was included as random effect, thus forming two level model^12^. Predictors included in the model were year, age, sex, insurance, income, Elixhauser comorbidity index, length of stay, hospital bed size, hospital teaching status, hospital ownership, and complications (mechanical, neurological, infectious, urinary, respiratory, cardiovascular, systemic, and surgical complications). Hospitalization costs were calculated by multiplying total hospital charges and cost-to-charge ratios. The costs for each year were adjusted according to the 2014 inflation levels based on the US Consumer Price Index. All tests were two tailed and statistical significance was set at *p* < 0.05.

## Results

A total of 181,545,077 hospitalizations were recorded during 2010–2014, and 44,846 index cases underwent resection for brain metastasis. Among them, 7,695 (17.8%) had unplanned readmissions within the first 30 days after discharge from the index hospitalization. The readmission rate did not change significantly during the five-year study period (*P for trend* = 0.286). Among patients who were readmitted, age-grouping was fairly unequal (18–39, 4.9%; 40–64, 55.1%; ≥ 65 years, 38.8%), but gender equally distributed (46.4% female, 53.6% male). The majority of readmitted patients were Medicare beneficiaries (42.5%) and private insurance (37.2%), followed by Medicaid (14.8%) and self-pay (1.9%). Patients were nearly equally distributed in all four household income quartiles. The majority of the patients were admitted in large (74.7%), private non-profit (75.9%), and metropolitan teaching (71.3%) hospitals. Table 1 shows the differences in demographic, hospital, and clinical characteristics for patients who did and did not experience unplanned readmissions within 30-days.

**Table 1:** Demographic, hospital, and clinical characteristics during principal admissions of patients undergoing resection for brain metastasis in the United States, 2010–2014.

Variables	Index cases	Unplanned 30-day readmission		*P* value
		Yes	No	
Total sample (unweighted)	18,888	3,304	15,584	
Total sample (weighted)	44,846	7,965	36,849	
**Age, % (SE)**				0.338
0–17 years	0.90% (0.12)	1.2% (0.23)	0.80% (0.13)	
18–39 years	4.6% (0.19)	4.9% (0.46)	4.6% (0.19)	
40–64 years	56.4% (0.50)	55.1% (1.1)	56.6% (0.52)	
≥ 65 years	38.1% (0.53)	38.8% (1.1)	38.0% (0.53)	
Female, % (SE)	51.8% (0.45)	46.4% (1.1)	53.0% (0.47)	< 0.001
**Primary payer, % (SE)**				< 0.001
Medicare	40.7% (0.53)	42.5% (1.1)	40.4% (0.54)	
Medicaid	13.3% (0.39)	14.8% (0.79)	12.9% (0.42)	
Private	39.7% (0.62)	37.2% (1.2)	40.1% (0.63)	
Self-pay	2.5% (0.15)	1.9% (0.27)	2.6% (0.17)	
No charge	0.20% (0.04)	0.20% (0.08)	0.20% (0.04)	
Other	3.7% (0.24)	3.4% (0.41)	3.8% (0.24)	
**Median household income for patient’s ZIP Code, % (SE)**				0.290
Quartile 1	25.8% (0.76)	27.4% (1.2)	25.4% (0.76)	
Quartile 2	25.0% (0.58)	24.5% (1.0)	25.1% (0.61)	
Quartile 3	25.0% (0.5)	24.7% (0.91)	25.1% (0.53)	
Quartile 4	24.1% (0.85)	23.3% (1.0)	24.4% (0.88)	
**Hospital bed size, % (SE)**				0.003
Small	6.1% (0.91)	7.9% (0.75)	6.1% (0.89)	
Medium	16.3% (0.88)	17.3% (1.1)	16.5% (0.88)	
Large	77.6% (1.2)	74.7% (1.3)	77.4% (1.2)	
**Teaching status, % (SE)**				< 0.001
Metropolitan non-teaching	19.4% (0.73)	24.0% (0.98)	19.5% (0.76)	
Metropolitan teaching	79.1% (0.77)	71.3% (1.1)	78.9% (0.79)	
Non-metropolitan hospital	1.5% (0.20)	4.6% (0.36)	1.6% (0.20)	
**Hospital ownership, % (SE)**				0.026
Government, nonfederal	15.3% (1.4)	13.9% (1.2)	15.2% (1.3)	
Private, not-profit	75.9% (1.5)	75.9% (1.6)	76.0% (1.5)	
Private, invest-own	8.8% (0.86)	10.3% (1.2)	8.8% (0.84)	
Elixhauser comorbidity index > 3, % (SE)	26.4% (0.49)	37.9% (1.2)	25% (0.49)	< 0.001
Length of stay ≥ 5 days, % (SE)	57.3% (0.70)	52% (1.0)	56.1% (0.72)	< 0.001
Death, % (SE)	–	8.7% (0.54)	–	–

**Figure 2:**
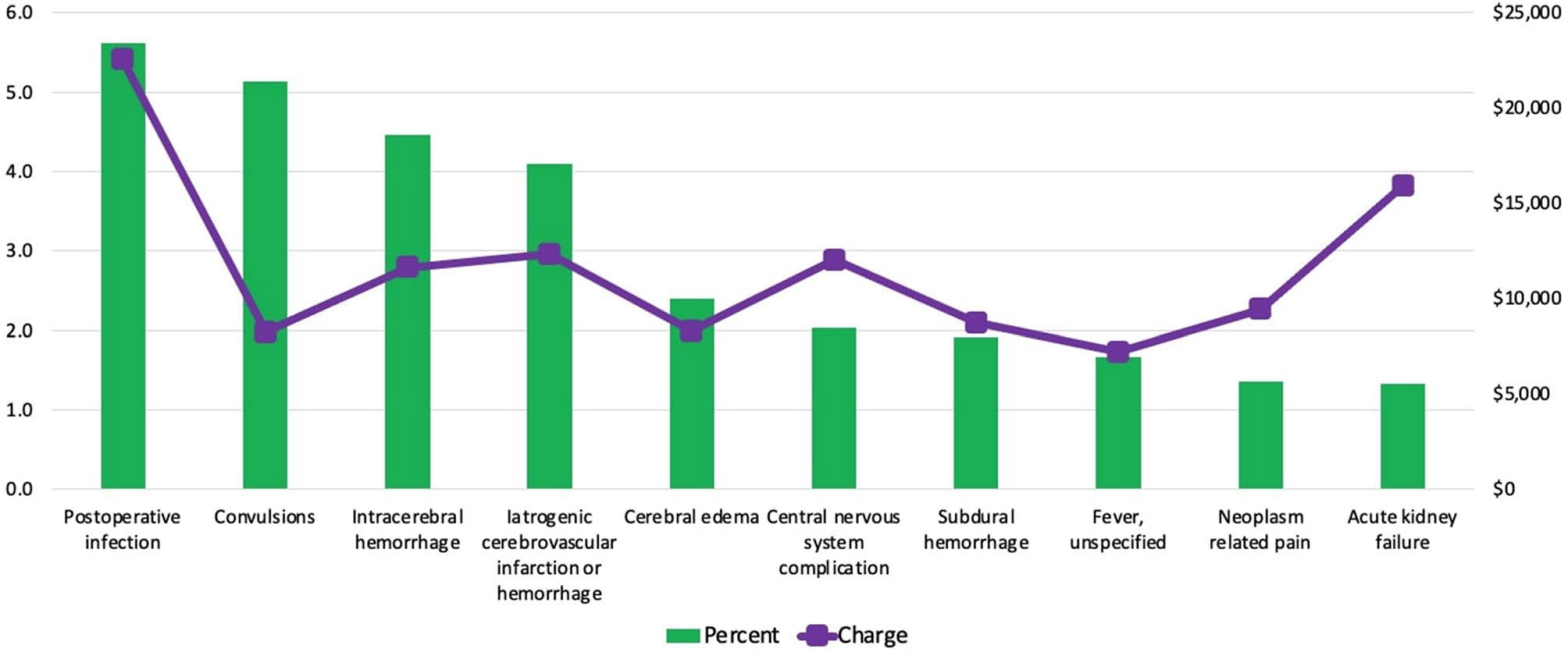
Primary reasons for readmissions by percent volume and median hospitalization charge following resection for brain metastasis.

The complications experienced during the index admission of patients undergoing resection for brain metastasis during the study period are shown in Table 2. The most common reason for readmission following craniotomy for brain metastasis was postoperative infection (5.6%), followed by convulsions (5.1%), intracerebral hemorrhage (4.5%), iatrogenic cerebrovascular infarction or hemorrhage (4.1%), and cerebral edema (2.4%) (Fig. 2). The total cost of unplanned 30-day readmissions following resection for brain metastasis was $132,096,474 during the study period, reflecting 49% of all national admissions data. Among the most common reasons for readmission, the costliest readmission was for postoperative infection ($22,534), followed by acute kidney failure ($15,919), iatrogenic cerebrovascular infarction or hemorrhage ($12,306), central nervous system complication ($12,040), and intracerebral hemorrhage ($11,626). Figure 2 illustrates the top 10 primary reasons for readmissions by percent volume and median hospitalization charge.

**Table 2:** Complications during principal admission of patients undergoing resection for brain metastasis in the United States, 2010–2014.

Complications	Unplanned 30-day readmission		*P* value
	Yes	No	
Mechanical wound, % (SE)	1.2% (0.19)	0.48% (0.07)	< 0.001
Neurological complications, % (SE)	10.2% (0.33)	8.8% (0.57)	0.035
Infection complications, % (SE)	7.1% (0.5)	0.39% (0.06)	< 0.001
Urinary complications, % (SE)	0.20% (0.15)	0.25% (0.07)	0.753
Pulmonary complications, % (SE)	1.3% (0.12)	0.61% (0.13)	< 0.001
Gastrointestinal complications, % (SE)	0.11% (0.05)	0.03% (0.01)	0.031
Cardiovascular complications, % (SE)	2.6% (0.4)	1.9% (0.14)	0.037
Systemic complications, % (SE)	0.41% (0.09)	0.18% (0.05)	0.015
Complications during the surgical procedure, % (SE)	0.28% (0.09)	0.49% (0.06)	0.086

Factors associated with 30-day readmission identified by multivariable hierarchical logistic regression are shown in Table 3. Results of the regression analysis showed that increasing age, male sex, having Medicaid (compared to Medicare), having higher Elixhauser comorbidity index, longer length of stay, admission to metropolitan teaching (compared to metropolitan non-teaching hospital), having neurological and infectious complications were associated with higher risk for 30-day readmission following discharge after an index admission for craniotomy for brain metastasis, while self-pay (compared to Medicare) and admission to non-metropolitan hospital (compared to metropolitan non-teaching hospital) were associated with lower risk 30-day readmission.

**Table 3:** Regression analysis showing factors associated with readmission following craniotomy for brain metastasis in the United States, 2010–2014.

Year	Odds ratio (95% CI)
2010	Reference
2011	0.984 (0.852–1.136)
2012	1.028 (0.885–1.194)
2013	0.929 (0.803–1.074)
2014	0.983 (0.853–1.133)
**Age category**	
0–17 years	Reference
18–39 years	0.82 (0.471–1.429)
40–64 years	1.555 (1.348–1.672)
≥ 65 years	1.786 (1.401–1.974)
Male	1.29 (1.172–1.419)
**Insurance**	
Medicare	Reference
Medicaid	1.513 (1.338–1.625)
No charge	0.713 (0.272–1.87)
Other pay	0.765 (0.565–1.035)
Private	0.918 (0.783–1.077)
Self-pay	0.587 (0.413–0.833)
**Income**	
Quartile 1	Reference
Quartile 2	0.947 (0.818–1.096)
Quartile 3	0.957 (0.832–1.100)
Quartile 4	0.929 (0.812–1.064)
Elixhauser comorbidity index > 3	1.374 (1.239–1.523)
Length of stay ≥ 5 days	1.215 (1.1–1.343)
**Hospital bed size**	
Small	Reference
Large	0.934 (0.796–1.095)
Medium	0.831 (0.685–1.009)
**Teaching status**	
Metropolitan non-teaching	Reference
Metropolitan teaching	1.291 (1.163–1.436)
Non-metropolitan hospital	0.872 (0.602–0.962)
**Hospital ownership**	
Government, nonfederal	Reference
Private, invest-own	1.042 (0.831–1.308)
Private, not-profit	0.984 (0.86–1.125)
Mechanical complications	0.781 (0.392–1.555)
Neurological complications	1.178 (1.027–1.35)
Infectious complications	1.447 (1.233–2.859)
Urinary complications	1.054 (0.447–2.488)
Respiratory complications	0.923 (0.664–1.283)
Cardiovascular complications	0.805 (0.586–1.106)
Systemic complications	0.908 (0.353–2.338)
Surgical complications	0.867 (0.492–1.528)

## Discussion

Quantifying the impact of readmissions after interventional or other procedures is an important part of quality improvement and assessment strategies. Prior studies using data from the Veterans Affairs Surgical Quality Improvement Program demonstrated that 30-day readmissions have trended downward in multiple surgical subspecialties, including neurosurgical procedures^13^. However, patients with brain metastasis represent a unique and understudied group of patients with additional challenges. Using a unique national data set, this study found that the 30-day rate of unplanned readmission following resection of a brain metastasis was approximately 17.8% and remained stagnant across the study period. The plateau in the rate of readmission is a major concern given the cost and morbidity impacts. To the best of our knowledge, this is the first study to look at unplanned readmissions for patients with brain metastases using a national data set. Identifying these risk factors and developing measures to prevent unplanned readmissions are key to reducing healthcare costs. We recognize that the data from AHRQ and HCUP have a lag between most recent available dataset to the current day. Though the post-surgical treatment and care paradigms have likely developed since 2014, there have been no recent studies or policy changes that have been implemented since that time period that would be expected to reduce the rate of readmissions appreciably beyond that described in the manuscript. Therefore, this highlights the importance of the work that, to the best of our knowledge, the readmission rate remained stagnant during the study period and would be expected to be representative of current day practice.

Our study found eight risk factors associated with an increase in 30-day unplanned readmission following resection of brain metastasis, including age, sex, primary payer, hospital teaching status, Elixhauser comorbidity index, length of stay, infectious complications, and neurosurgical complications. Older age, insurance status, and presence of multiple comorbidities are key features associated with unplanned readmissions across different surgical series. For example, Caplan et al. demonstrated that in a single, multi-hospital, academic medical center, publicly insured supratentorial craniotomy patients were twice as likely to get readmitted as those with private insurance^14^. While we do not know for certain the mechanism as to why public insurance is significantly associated with an increased rate of readmission, it may in part be due to better and more sustainable access to health care facilities for patients with private insurance^15^. Medicaid and Medicare patients are also among the sicker and older patients with several comorbidities, which may result in recurrent hospitalizations regardless of the prior interventions^15^. Previous studies also demonstrated private insurance patients have lower in-hospital mortality and better discharge disposition^16^, while those on government sponsored insurance are more likely to get readmitted^3^. Particularly, Dohono et al. reported that two or more comorbidities increased the chance of readmission by 1.26 times^17^. Similarly, in the present study we found that patients with > 3 comorbidities had a significantly higher risk of readmission. We also found that length of stay of 5 days or more significantly increased the rate of readmission compared to patients with a length of stay < 5 days. Other series also demonstrated the association of hospital stay with risk of unplanned readmission, whether the extended stay is due to medical, surgical, or social issues^18^. Unlike previous studies that identified one or a few variables associated with risk of readmission, the study generated a novel model to stratify patients into risk categories. Taken together, patients in the “high” risk category (i.e., elderly patients with multiple comorbidities) may warrant extra measures to ensure appropriate discharge interventions are performed to prevent unplanned readmission.

Unplanned readmission after surgery is a key metric of interest as it correlates with a 30% increase in the risk of mortality and represents a significant healthcare burden to society^19^. In 2004, the Centers of Medicare and Medicaid Services reported that unplanned hospital readmissions totaled $17.4 billion in costs to Medicare system alone, leading to the institution of a 1% payment penalty for hospitals with excessive unplanned readmissions^20^. In this study, we found the most common and costly complication following 30-day readmission among patients undergoing craniotomy for brain metastasis was post-operative infection. Our analysis showed the cost for 30-day unplanned readmission for patients with brain metastasis was $26.4 million per year. Due to this substantial healthcare expenditure, factors associated with readmissions must be identified and targeted to decrease the financial burden.

Although the NRD was specifically designed to allow for readmission analysis, there are several limitations to this dataset and analyses. First, this is an administrative database and, therefore, limited in terms of the available clinical variables that then limits development of a comprehensive risk-prediction model. Future studies should include large datasets with increased patient-level granularity to further refine prediction models. Second, the NRD links each admission to a state-specific identifier. Hence, a patient admitted to a state (index admission) for resection, but readmitted to another state for a complication is not be captured in this database. This would underestimate the rates of readmissions. However, we do assume that these underestimates are proportional in each of the risk categories.

In the United States, approximately 1 in 6 patients who undergoes craniotomy of a brain metastasis will experience an unplanned readmission within 30 days. Complications are not only common, but also costly. Therefore, hospital systems should consider the identified key risk factors associated with unplanned readmissions and develop strategies to risk-stratify patients and provide dedicated interventions to reduce the rates of readmission and improve the quality of care we deliver.

## Supplementary Information

Supplementary Information.

## References

[1] Suh JH (2020). Current approaches to the management of brain metastases. Nat. Rev. Clin. Oncol..

[2] Kotecha R, Gondi V, Ahluwalia MS, Brastianos PK, Mehta MP (2018). Recent advances in managing brain metastasis. F1000Research.

[3] Marcus LP (2014). Incidence and predictors of 30-day readmission for patients discharged home after craniotomy for malignant supratentorial tumors in California (1995–2010). J. Neurosurg..

[4] Jencks SF, Williams MV, Coleman EA (2009). Rehospitalizations among patients in the Medicare fee-for-service program. N. Engl. J. Med..

[5] Centers for Medicare and Medicaid Services (CMS), H. Medicare program; hospital inpatient prospective payment systems for acute care hospitals and the long-term care hospital prospective payment system and fiscal year 2013 rates; hospitals’ resident caps for graduate medical education payment purposes; qual. *Fed. Regist.* **77**, 53257–750 (2012). 22937544

[6] Zuckerman RB, Sheingold SH, Orav EJ, Ruhter J, Epstein AM (2016). Readmissions, observation, and the hospital readmissions reduction program. N. Engl. J. Med..

[7] Gupta A (2018). Association of the hospital readmissions reduction program implementation with readmission and mortality outcomes in heart failure. JAMA Cardiol..

[8] Jacobs DM (2018). Early hospital readmissions after an acute exacerbation of chronic obstructive pulmonary disease in the nationwide readmissions database. Ann. Am. Thorac. Soc..

[9] Bailey KL (2019). Short-term readmissions after open, thoracoscopic, and robotic lobectomy for lung cancer based on the nationwide readmissions database. World J. Surg..

[10] Horwitz LI (2014). Development and use of an administrative claims measure for profiling hospital-wide performance on 30-day unplanned readmission. Ann. Intern. Med..

[11] Vandenbroucke JP (2014). Strengthening the reporting of observational studies in epidemiology (STROBE): Explanation and elaboration. Int. J. Surg..

[12] Arora S (2017). Etiologies, trends, and predictors of 30-day readmission in patients with heart failure. Am. J. Cardiol..

[13] Han S, Smith TS, Gunnar W (2014). Descriptive analysis of 30-day readmission after inpatient surgery discharge in the Veterans Health Administration. JAMA Surg..

[14] Caplan IF (2019). The LACE+ index fails to predict 30–90 day readmission for supratentorial craniotomy patients: A retrospective series of 238 surgical procedures. Clin. Neurol. Neurosurg..

[15] Sommers BD, Gawande AA, Baicker K (2017). Health insurance coverage and health—what the recent evidence tells us. N. Engl. J. Med..

[16] Barker FG (2004). Craniotomy for the resection of metastatic brain tumors in the U.S., 1988–2000: decreasing mortality and the effect of provider caseload. Cancer.

[17] Donoho DA (2018). Predictors of 30- and 90-day readmission following craniotomy for malignant brain tumors: analysis of nationwide data. J. Neurooncol..

[18] Adogwa O (2016). Racial disparities in 30-day readmission rates after elective spine surgery: a single institutional experience. Spine.

[19] Schipmann S (2020). The 30-day readmission rate in neurosurgery-a useful indicator for quality assessment?. Acta Neurochir. (Wien).

[20] Dickinson H (2015). Unplanned readmissions and survival following brain tumor surgery. J. Neurosurg..

