# Supplementary material for: Factors associated with unplanned readmissions and costs following resection of brain metastases in the United States
Source: Sci Rep. 2021 Nov 12;11:22152. doi: 10.1038/s41598-021-01641-4 (PMC8589950; doi:10.1038/s41598-021-01641-4)
Supplement: Supplementary file 1 — Supplementary Information. [file 41598_2021_1641_MOESM1_ESM.docx]

Supplementary Table 1. ICD-9-CM diagnosis and procedure codes

| **Condition** | **ICD-9-CM Diagnosis and Procedure Codes** |
| --- | --- |
| Brain metastasis | 1983 |
| Resection | 0159 |
| Mechanical wound | 99883, 99812, 99813, 9986, 9983 |
| Neurological complications | 99702, 99811, 99812, 431, 4329, 43491 |
| Infection complications | 9985, 99859, 99851 |
| Urinary complications | 9975 |
| Pulmonary complications | 9973, 5185, 5121, 5185, 5184 |
| Gastrointestinal complications | 9974 |
| Cardiovascular complications | 99779, 41511, 99702, 9972, 9971 |
| Systemic complications | 9980, 99889 |
| Complications during the surgical procedure | 9982, 9984, 99811 |
